# Metabolic syndrome is associated with breast cancer mortality: A systematic review and meta‐analysis

**DOI:** 10.1111/joim.20052

**Published:** 2025-01-08

**Authors:** Sixten Harborg, Helene Borup Larsen, Stine Elsgaard, Signe Borgquist

**Affiliations:** ^1^ Department of Oncology Aarhus University Hospital/Aarhus University Aarhus Denmark; ^2^ Department of Clinical Epidemiology Aarhus University Hospital/Aarhus University Aarhus Denmark; ^3^ Department of Clinical Sciences Lund, Oncology Lund University Lund Sweden

**Keywords:** breast neoplasms, meta‐analysis, metabolic syndrome, mortality, recurrence, review

## Abstract

**Background:**

This systematic review and meta‐analysis assesses the association between metabolic syndrome and breast cancer (BC) outcomes in BC survivors.

**Methods:**

Systematic searches were carried out in PubMed and Embase using variations of the search terms: breast neoplasms (population), metabolic syndrome (exposure), and survival (outcome). Metabolic syndrome was characterized according to the American Heart Association, which includes the presence of three out of five abnormal findings among the risk factors: high blood pressure, high triglycerides, low high‐density lipoprotein, high fasting glucose, and central obesity. Data were obtained from observational studies and randomized controlled trials that utilized survival statistics and reported survival ratios to investigate how the presence of metabolic syndrome at the time of BC diagnosis is associated with BC outcomes. Study data were independently extracted by two authors, and effect sizes were pooled using random‐effects models.

**Results:**

From the 1019 studies identified in the literature search, 17 were deemed eligible. These encompassed 42,135 BC survivors. The pooled estimates revealed that BC survivors who had metabolic syndrome at the time of their BC diagnosis experienced increased risk of recurrence (HR 1.69, 95% CI: 1.39–2.06), BC mortality (HR 1.83, 95% CI: 1.35–2.49), and shorter disease‐free survival (HR 1.57, 95% CI: 1.36–1.81) compared to BC survivors without metabolic syndrome.

**Conclusions:**

Among BC survivors, metabolic syndrome was associated with inferior BC outcomes. This necessitates the creation of clinical guidelines that include metabolic screening for BC survivors. Further research should identify effective interventions to reduce the prevalence of metabolic syndrome among BC survivors to improve metabolic health and BC outcomes.

## Graphical abstract

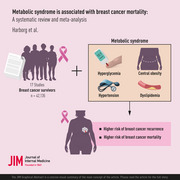

**Abbreviations**
BC: breast cancer
ER: estrogen receptor
HR: hazard ratio
CI: confidence interval
ES: effect size
DFS: disease‐free survival
OS: overall survival
PRISMA: Preferred Reporting Items for Systematic Reviews and Meta‐Analysis
PICO: population, intervention, comparison, outcome

## Introduction

Metabolic syndrome, a collection of risk factors that escalate the probability of developing cardiovascular and chronic diseases, including myocardial infarction, stroke [1], and type 2 diabetes [2], has also been associated with a poor prognosis in breast cancer (BC) [3]. These risk factors encompass high blood pressure, high fasting blood sugar, excess body fat around the waist, and abnormal lipid levels [4].

**Fig. 1:**
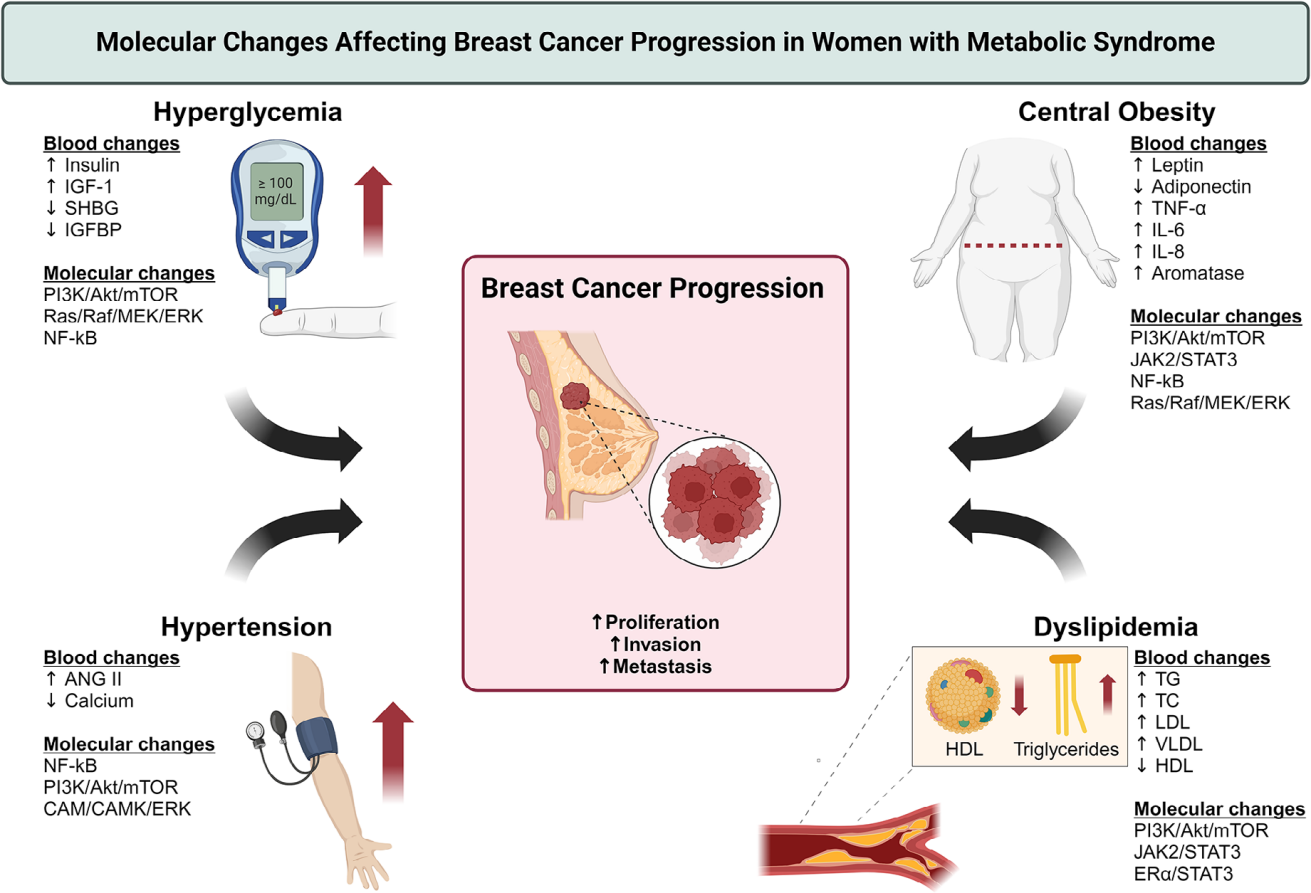
Molecular changes affecting breast cancer progression in women with metabolic syndrome. ANG II, angiotensin II; CAM/CAMK/ERK, calcium–calmodulin (CAM)/calcium‐calmodulin‐dependent protein kinase (CAMK)/extracellular signal‐regulated kinase (ERK); ERα/STAT3, estrogen receptor alpha/signal transducer and activator of transcription 3; HDL, high‐density lipoprotein; IGF‐1, insulin growth factor 1; IGFBP, insulin‐like growth factor‐binding protein; IL‐6, interleukin 6; IL‐8, interleukin 8; JAK2/STAT3, Janus kinase 2/signal transducer and activator of transcription 3; LDL, low‐density lipoprotein; NF‐kB, nuclear factor kappa‐light‐chain‐enhancer of activated B cells; PI3K/Akt/mTOR, phosphoinositide 3‐kinase/protein kinase B (Akt)/mammalian target of rapamycin; Ras/Raf/MEK/ERK, Ras/rapidly accelerated fibrosarcoma (Raf)/mitogen‐activated protein kinase (MEK)/extracellular signal‐regulated kinase (ERK); SHBG, sex‐hormone‐binding‐globulin; TC, total cholesterol; TG, triglycerides; TNF‐α, tumor necrosis factor alpha; VLDL, very low‐density lipoprotein. Source: Figure created by the authors using BioRender (www.biorender.com).

Studies have shown that women with metabolic syndrome are at a higher risk of developing BC [5], and their prognosis following a BC diagnosis tends to be poorer [3]. The precise mechanisms through which metabolic syndrome heightens the risk of BC and its recurrence remain unclear but are believed to be linked to chronic inflammation and hormonal imbalances (Fig. 1). One possible explanation posits that the excessive body fat associated with metabolic syndrome results in increased levels of estrogen, which may stimulate the growth of estrogen receptor (ER)‐positive BC cells [6, 7, 8]. This excess body fat may also induce changes in the tumor microenvironment, potentially promoting the development of BC metastases [9, 10, 11]. Moreover, the chronic inflammation associated with metabolic syndrome might foster cancer cell growth, and due to a compromised immune system, prevent the body from efficiently combating cancer development [12].

Metabolic syndrome is a collection of potentially modifiable risk factors that can be improved by lifestyle changes and/or medical intervention. Lifestyle changes, such as a healthy diet and regular physical activity, can induce weight loss [13, 14, 15, 16]. These can be supplemented with medical treatments like cholesterol‐lowering drugs, antihypertensive medication, antidiabetic medication, and anti‐obesity medication [17]. Therefore, an initially poorer BC prognosis in these women could potentially be improved through lifestyle modifications [18] and/or medical treatment [19, 20, 21, 22, 23].

This study aimed to assess the possible negative effects of metabolic syndrome on BC prognosis. We hypothesized that the presence of metabolic syndrome would be associated with poorer cancer‐specific outcomes in women with a diagnosis of BC.

## Methods

The current review was preregistered with PROSPERO (*Reg. No.: CRD42024526395*) and is reported in compliance with the PRISMA (Preferred Reporting Items for Systematic Reviews and Meta‐Analysis) guidelines.

### Data sources and search strategy

Systematic searches were conducted in PubMed and Embase using variations of the search terms “breast neoplasms” (population), “metabolic syndrome” (exposure), and “survival” (outcome). Searches were conducted up until June 21, 2024. The full search string is available in Table S1. Data were extracted from observational studies and randomized controlled trials using survival statistics and reported ratios of survival, for example, hazard ratios (HRs) or survival curves, to examine how the presence of metabolic syndrome at the time of diagnosis of BC is associated with BC outcomes. Following the PRISMA guidelines, studies were screened, and data were independently extracted by two authors (SH, HBL). Any disagreements were resolved through consensus or by involving a third reviewer (SB).

### Selection criteria and data extraction

Based on the PICO approach (population, intervention/exposure, comparison, outcome), we established the following inclusion and exclusion criteria. The population was delineated as women aged 18 years and older with breast neoplasm. Exposure was defined by the presence of metabolic syndrome, comparators were established as the presence or absence of metabolic syndrome, and outcomes were denoted as all endpoints reporting events related to BC survival (i.e., any recurrence, distant metastases, or death). We placed no constraints on publication year, geographical circumstances, or follow‐up period. Gray literature, such as conference abstracts and unpublished studies, was not considered. Inclusion was restricted to English‐language publications only. Two authors (SH and HBL) independently screened titles and abstracts using Covidence systematic review software (www.covidence.org). After studies were excluded based on titles and abstracts, the remaining full‐text references underwent review. Any disagreements were discussed until consensus was achieved, or a third author (SB) was consulted to resolve. Data were extracted by two authors (SH and HBL), validated and then classified according to predetermined characteristics, including study name, patient characteristics, treatment characteristics, exposure (metabolic syndrome), and outcome data (recurrence, BC mortality, disease‐free survival [DFS], or overall survival [OS]).

### Definition of metabolic syndrome

Exposure was defined as the presence of metabolic syndrome, which is having three or more out of five metabolic risk factors. The definition does not distinguish the number or type of risk factors present. The threshold for metabolic syndrome exposure was established based on the harmonized metabolic syndrome definition from the American Heart Association [4]. Conversely, the absence of metabolic syndrome was defined as having fewer than three risk factors. For statistical analysis, women were divided into two groups based on the presence or absence of metabolic syndrome, and these groups were compared.

### Endpoints and their definitions

The primary endpoint of this meta‐analysis was DFS, defined as the period from BC diagnosis to the first BC event, such as recurrence, or death. Whenever possible, DFS was the favored effect estimate derived from the included studies. If DFS data were unavailable, we initially tried to extract effect estimates of recurrence, followed by BC mortality. If neither was available, we integrated effect estimates of OS into the DFS estimate. Secondary endpoints in the study consisted of recurrence (designated as the time from BC diagnosis to its recurrence), BC mortality, and OS (specified as the interval from BC diagnosis to death, irrespective of cause).

### Analytical strategy

Observational cohort studies and clinical trials, which analyzed data either prospectively or retrospectively, were reviewed and subjected to meta‐analysis to calculate the pooled overall effect estimate and its precision.

### Pooling effect sizes

An inverse variance‐weighted random‐effects model, considering the precision of each study, was applied to all analyses. HRs larger than 1.0 indicated an effect in the hypothesized direction, that is, metabolic syndrome is associated with a shorter DFS. When studies reported effect estimates on metabolic syndrome as stratified analyses by exposures other than metabolic syndrome, the estimates for each stratum were combined into one. The individual and pooled HRs, along with their associated 95% confidence intervals (CI), are presented in forest plots. Estimates were considered statistically associated if the CIs did not cross 1.0. Heterogeneity was investigated using *Q* and *I* ^2^ statistics. All statistical analyses were conducted with Stata version 18.

### Publication bias

The potential for publication bias was evaluated using funnel plots (Figs. S1–S8) and Egger's test [24]. If the results suggested possible publication bias, we conducted sensitivity analyses by imputing the “missing studies” and calculating adjusted effect estimates using the Duval and Tweedie trim‐and‐fill method (Table S1 and Fig. S9)[25].

### Moderator analyses

We examined potential sources of heterogeneity (*I* ^2^ > 0.0) by conducting meta‐regression based on random‐effects models and using the maximum likelihood method. We considered three probable moderators on the effect size (ES): the median follow‐up time in months, the studies’ mean age of women, and the population size of the studies.

## Results

### Study population

**Fig. 2:**
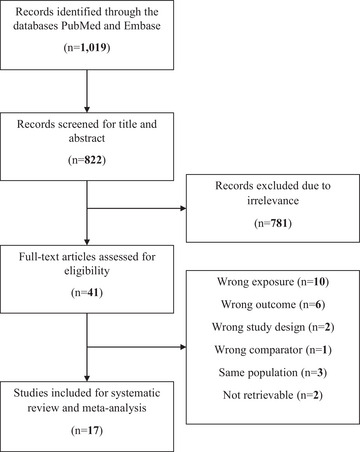
PRISMA (Preferred Reporting Items for Systematic Reviews and Meta‐Analysis) flow diagram of selected studies.

The systematic literature search identified a total of 1019 studies. After removing duplicates, screening titles, and abstracts, 41 studies qualified for full‐text review and 17 studies were ultimately selected for inclusion in the data analysis. Please refer to Fig. 2 for the study selection process.

The 17 independent studies considered for the analysis involved 42,135 BC survivors. Seven studies, which included 9029 women, offered data on BC recurrence, another 7 studies involving 31,008 women reported on BC mortality, and 8 studies with 17,235 women provided data on OS. The characteristics of the studies are listed in Table 1. The average age was 53.2 years, and the median time for follow‐up fluctuated between 33.6 and 278.4 months, with an average follow‐up time of 94.8 months.

**Table 1:** Study characteristics of included studies.

Study (years published)	Country	Study period	Population	Menopausal status	Median follow‐up months (years)	Mean age at diagnosis (years)	ES for disease‐free survival (95% CI)	ES for overall survival (95% CI)	ES for breast cancer mortality (95% CI)	ES for breast cancer recurrence (95% CI)
Pasanisi (2006) [31]	Italy	N/R	110	Postmenopausal	66 (5.5)	56.8				3.0 (1.2, 7.1)
Bjørge (2010) [26]	Norway, Sweden, Austria	1974–2005	4862	Pre‐ and postmenopausal	132 (11.0)	58			1.18 (1.03, 1.32)	
Oh (2011) [30]	Korea	2001–2004	747	Pre‐ and postmenopausal	62.2 (5.2)	45.9				1.87 (0.77, 4.51)
Berrino (2014) [27]	Italy	2008–2013	2092	Pre‐ and postmenopausal	33.6 (2.8)	51.4				2.17 (1.31, 3.60)
Calip (2014) [29]	USA	1990–2011	4216	Pre‐ and postmenopausal	75.6 (6.3)	63	1.50 (1.08, 2.07)	1.81 (1.44, 2.29)	1.65 (1.02, 2.69)	1.65 (1.15, 2.38)
Fan (2015) [32]	China	2004–2008	1249	N/R	79 (6.6)	49		0.72 (0.39, 1.34)		1.01 (0.56, 1.82)
Cho (2018) [33]	Korea	1996–2003	5668	N/R	N/R	50	1.59 (1.06, 2.41)	2.67 (1.49, 4.79)		
Gathirua‐Mwangi (2018) [34]	USA	1988–1994	10,014	N/R	46.4 (3.9)	55.5			2.24 (1.34, 3.75)	
Grybach (2018) [35]	Ukraine	2009–2015	202	Pre‐ and postmenopausal	N/R	N/R		1.72 (0.90, 3.31)		
Dibaba (2019) [36]	USA	1995–N/R	6631	Pre‐ and postmenopausal	168 (14)	N/R			2.02 (1.29, 3.17)	
Watanabe (2019) [28]	Japan	1992–2013	6	N/R	N/R	N/R			11.90 (2.25–62.89)	
Buono (2020) [37]	Italy	2009–2013	717	Pre‐ and postmenopausal	85.2 (7.1)	N/R	1.51 (0.96, 2.38)	3.01 (1.72, 5.28)	3.16 (1.64, 6.07)	
Kennard (2021) [38]	USA	2007–2013	177	N/R	69.6 (5.8)	59.9	2.24 (1.08, 4.63)	1.92 (0.88, 4.21)		2.50 (1.20, 5.18)
Taroeno‐Hariadi (2022) [41]	Indonesia	2010–2020	223	Pre‐ and postmenopausal	51 (4.3)	49	1.03 (0.63, 1.69)	1.44 (0.58–3.57)		
Yang (2022) [39]	China	2010–2020	438	Pre‐ and postmenopausal	109.3 (9.1)	47.4				1.51 (1.05, 2.19)
Zhou (2023) [40]	China	2012–2022	221	Pre‐ and postmenopausal	71 (6)	N/R	2.23 (1.25, 3.97)	2.59 (1.36, 4.92)		
Chlebowski (2024) [55]	USA	1993–2020	4562	Postmenopausal	278.4 (23.2)	N/R		1.53 (1.26–1.85)	1.44 (1.02–2.04)	

Abbreviations: ES, effect size; N/R, not retrievable.

### Association between metabolic syndrome and breast cancer outcomes

**Fig. 3:**
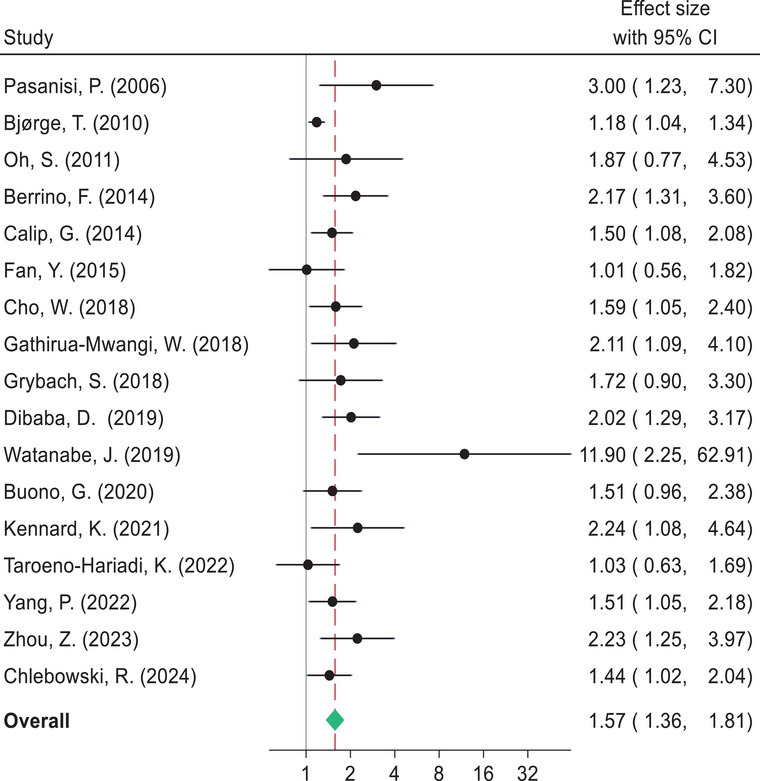
Forest plot comparing disease‐free survival for breast cancer survivors with metabolic syndrome and without metabolic syndrome.

**Fig. 4:**
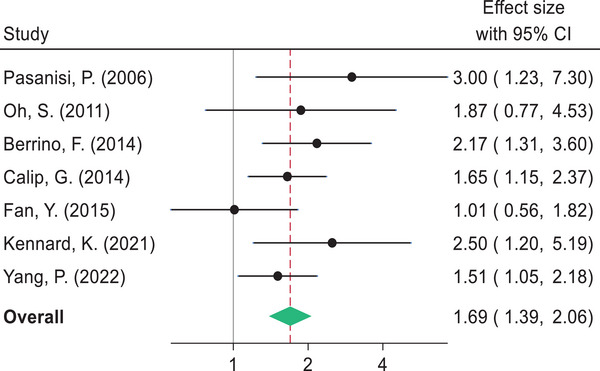
Forest plot comparing breast cancer recurrence for breast cancer survivors with metabolic syndrome and without metabolic syndrome.

**Fig. 5:**
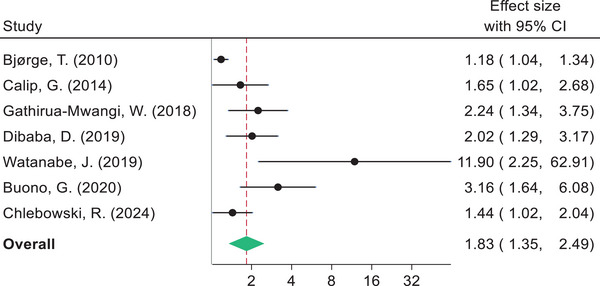
Forest plot comparing breast cancer mortality for breast cancer survivors with metabolic syndrome and without metabolic syndrome.

**Fig. 6:**
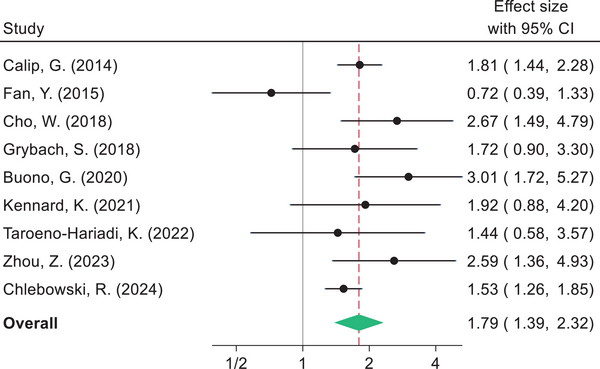
Forest plot comparing overall survival for breast cancer survivors with metabolic syndrome and without metabolic syndrome.

The pooled estimates indicate that BC survivors diagnosed with metabolic syndrome had shorter DFS compared to women without metabolic syndrome (HR 1.57, 95% CI: 1.36–1.81). Likewise, women with metabolic syndrome had a higher risk of BC recurrence (HR 1.69, 95% CI: 1.39–2.06), BC mortality (HR 1.83, 95% CI: 1.35–2.49), and shorter OS (HR 1.79, 95% CI: 1.39–2.32) compared to those without metabolic syndrome (Figs. 3, 4, 5, 6).

### Sensitivity analyses

**Fig. 7:**
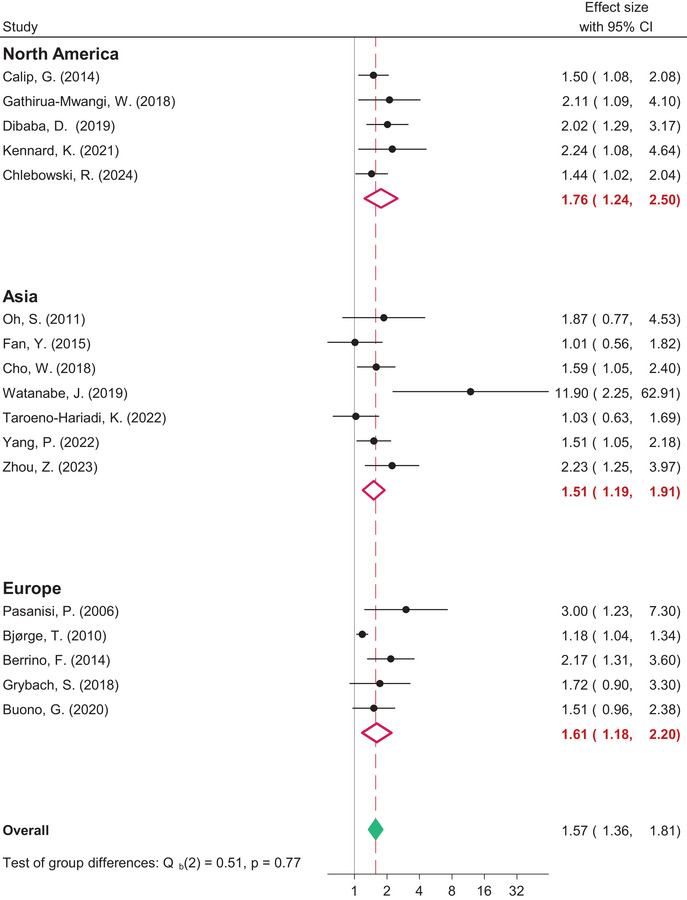
Forest plot comparing disease‐free survival for breast cancer survivors with metabolic syndrome and without metabolic syndrome according to continent of study execution.

We conducted several sensitivity analyses to study the moderators of the association. First, we explored whether the study's population size, follow‐up time, or median age moderated the relationship between metabolic syndrome and inferior BC outcome. However, no interaction was found between the possible moderators and the association. Second, we examined the association between metabolic syndrome and DFS in cohorts comprising only postmenopausal women. We found a somewhat stronger association between metabolic syndrome and DFS (HR 1.80, 95% CI: 1.27–2.54) in subgroup analyses of postmenopausal women with BC compared to analyses of the full pooled population. Finally, to investigate any potential differences in the association between metabolic syndrome and BC outcome according to geographical location, we conducted distinct meta‐analyses based on the origin continent of the included studies. The association between metabolic syndrome and shorter DFS was consistent across all continents evaluated in the study. The association was nearly identical for North America (HR 1.66, 95% CI: 1.37–2.01), Asia (HR 1.51, 95% CI: 1.19–1.91), and Europe (HR 1.61, 95% CI: 1.16–2.20), as depicted in Fig. 7.

## Discussion

This study demonstrates that women living with metabolic syndrome when concurrently diagnosed with BC are at a higher risk of recurrence or death from their BC disease compared to women who do not have metabolic syndrome at diagnosis. In this systematic review and meta‐analysis, BC survivors with metabolic syndrome were found to be 57% more likely to experience a BC‐related event during follow‐up; likewise, they were 69% more susceptible to a recurrence of their BC, and 83% more likely to die from their BC than their counterparts without metabolic syndrome.

The study underscores an emerging health issue: the increasing worldwide incidence and prevalence of obesity, diabetes mellitus, and cardiovascular disease alongside the growing number of cancer survivors. We need measures aimed at impeding a poor prognosis for BC survivors and optimizing care to alleviate the burden of metabolic syndrome is an existing demand [18]. Cardiovascular mortality is the second leading cause of death among BC survivors [42], an issue that could also be tackled through treatments seeking to reverse metabolic syndrome. Current research suggests a less favorable prognosis for BC survivors struggling with concurrent diseases related to dyslipidemia [43] and diabetes [44, 45, 46]. At present, there are no guidelines recommending metabolic screening and interventions for BC survivors during and after their treatment course. Based on our findings and others, we propose that research is warranted into whether BC survivors might benefit from metabolic screening during their treatment course to improve their chances of avoiding a recurrence or death from their malignancy.

One of the primary benefits of metabolic screening in BC survivors is the early detection of comorbid conditions such as hypertension, diabetes, and dyslipidemia. Early identification allows for timely interventions that can enhance overall health and potentially improve cancer treatment outcomes. The comprehensive metabolic profiles achieved from metabolic screening could enable clinicians to more effectively tailor treatment plans. For example, managing hyperglycemia or dyslipidemia concurrent with cancer treatment can augment the effectiveness of cancer therapies and elevate women's overall quality of life [47]. Personalized treatment methods, inclusive of metabolic profiles, can result in better outcomes and fewer complications [48].

Metabolic screening also enhances patient awareness regarding the importance of managing their metabolic health, leading to improved adherence to preventive measures such as dietary changes, regular physical activity, and consistent monitoring of metabolic parameters [49]. These lifestyle adjustments can significantly mitigate the risk of metabolic syndrome [50] and its adverse effect on cancer prognosis [18].

Furthermore, with the increasing emergence of obesity, diabetes, and cardiovascular diseases among cancer survivors, metabolic screening may enhance long‐term management [51]. By tackling metabolic risk factors, healthcare providers may decrease the burden of these conditions and improve the overall health and survival of BC survivors. This proactive method to manage metabolic health guarantees that cancer survivors experience an improved quality of life and enhanced outcomes posttreatment.

Previous studies by Li et al. [3] and Dong et al. [52] assessed the relationship between metabolic syndrome and survival in BC survivors. Their results were similar to our study's conclusions, but they included fewer participants (*N* = 17,892). As such, they could not demonstrate the negative impact of metabolic syndrome on BC survival across outcomes. In contrast to our study, the one by Li et al. [3] found no association between metabolic syndrome and BC survival among Asian women. However, our study showed that women with BC and metabolic syndrome generally have a poorer prognosis, irrespective of their geographical location (be it North America, Asia, or Europe).

Similar to previous studies, we could not assess the association between metabolic syndrome and ER status. The studies included in our meta‐analysis did not consistently report ER status, which prevented us from conducting a stratified analysis. It is worth noting, however, that ER‐positive and ER‐negative BCs may have different associations with metabolic syndrome. Although estrogen levels are linked to the development of ER‐positive BC, components of metabolic syndrome like insulin resistance and inflammation might play more significant roles in ER‐negative tumors. Future studies should be stratified by ER status for a better understanding of these associations. Additionally, the data collected did not permit us to present survival estimates for each component of the metabolic syndrome. Previous research has shown that although all components of the metabolic syndrome are individually associated with all‐cause mortality and cardiovascular mortality, only obesity is established as a risk factor for BC mortality [53, 54] and may potentially drive the association [55].

The strength of this study lies in its comprehensive meta‐analytical approach, incorporating data from a large sample size of 42,135 BC survivors. This enhances the robustness and generalizability of the findings. The inclusion of diverse study populations from different geographical locations and healthcare settings further amplifies the applicability of the results. However, there are limitations to this study. There was heterogeneity among the included studies regarding the characteristics of the population and the follow‐up periods, which could potentially affect the consistency and comparability of the results. However, the moderator analyses did not indicate any interaction between these variables and the observed association. The potential for publication bias remains, as studies with significant findings are more likely to be published, potentially skewing the results. One particular study [56] with a small sample size had an ES with extreme values. Yet, the leave‐one‐out meta‐analysis [57] showed a consistent association and did not identify any studies that significantly attenuated the association. Furthermore, the analysis using the Duval and Tweedie trim‐and‐fill method to calculate adjusted effect estimates, including imputed “missing studies,” did not weaken the association. The discrepancy between risks of DFS and BC mortality might be due to the broad definition of DFS in this study, encompassing both cancer recurrence and death from any cause. This can introduce non‐cancer‐related factors into the analysis. Patients with metabolic syndrome have a higher risk of death due to comorbidities such as cardiovascular disease and diabetes. This broader definition likely diluted the association between metabolic syndrome and DFS, whereas BC mortality—which is more directly associated with cancer recurrence—would likely be less influenced by non‐cancer comorbidities. Thus, the risk of BC mortality may appear disproportionately higher compared to the broader outcome of DFS. Detailed information about the medications commonly prescribed for metabolic conditions (e.g., antihypertensive drugs, statins, and antidiabetic medications) was notably missing from the included studies. Pharmacological treatments, including drugs that target disease‐components of the metabolic syndrome, may influence metabolic pathways and/or cancer‐related processes, potentially confounding the observed associations between metabolic syndrome and BC outcomes. As individual studies in this meta‐analysis did not consistently account for medication usage, we were unable to control for the effects of these drugs across all studies. Additionally, our study lacks detailed subgroup analyses based on factors such as BC subtypes and treatment modalities. This would provide more profound insights into how metabolic syndrome affects different patient groups. Although the included studies adjusted for several known confounders, the possibility of residual confounding cannot be excluded. Factors such as genetic predispositions, lifestyle, and socioeconomic position, which were not examined, could partially account for the observed associations between metabolic syndrome and BC outcomes. The significance of the observed risks suggests that further study is required to better control for potential confounders. Lastly, because most included studies were observational, it limits the potential to establish causality between metabolic syndrome and BC outcomes.

Effective management of metabolic syndrome in BC survivors necessitates a comprehensive approach, including lifestyle modifications and pharmacological interventions. Lifestyle adjustments, the foundation for management, should encompass dietary alterations, heightened physical activity, and weight control. A diet enriched with fruits, vegetables, whole grains, and lean proteins along with regular exercise can assist in moderating the components of metabolic syndrome [58]. Pharmacological interventions are also essential. For instance, statins are used to manage dyslipidemia, antihypertensive medications control blood pressure, antidiabetic drugs manage blood glucose levels, and anti‐obesity medication handles excess adipose tissue depots [17]. It is evidenced that these interventions can positively influence cancer outcomes by forging an unfavorable environment for tumor progression [59]. For instance, statins, a common cholesterol‐lowering medication, have been shown to have anti‐tumorigenic effects in BC [20, 60]. It is crucial to further investigate several areas to better understand and manage the relationship between metabolic syndrome and BC outcomes. Longitudinal studies are required to evaluate the long‐term effect of managing metabolic syndrome on BC survival. Such studies could provide insights into how early and sustained intervention may influence recurrence and mortality rates over extended periods.

Randomized controlled trials should be conducted to test specific interventions targeting the components of metabolic syndrome in BC survivors. These trials could investigate the efficacy of various dietary patterns, exercise regimens, and pharmacological treatments to improve cancer outcomes [59]. Research should also delve into the genetic and molecular mechanisms underlying the association between metabolic syndrome and BC.

Cost‐effectiveness studies are vital for informing healthcare policy decisions. Many proposed interventions to reverse metabolic syndrome are inexpensive compared to the rising costs of new oncological treatments [61]. Hence, the suggested clinical intervention studies should aim to assess the economic impact of implementing regular metabolic screening and intervention programs in BC care, balancing the costs with potential benefits such as improved BC outcomes and quality of life.

## Conclusions

Among BC survivors, metabolic syndrome was associated with poorer BC outcomes. The findings of this study emphasize the importance of metabolic screening for BC survivors. Future research should focus on assessing how lipid control, reversing diabetes, and making healthy lifestyle choices could decrease the prevalence of metabolic syndrome in this population and ultimately enhance BC survival.

## Author contributions

**Sixten Harborg**: Conceptualization; methodology; formal analysis; data curation; writing—original draft; writing—review and editing; visualization; project administration. **Helene Borup Larsen**: Data curation; writing—review and editing. **Stine Elsgaard**: Data curation; writing—review and editing. **Signe Borgquist**: Conceptualization; methodology; writing—review and editing; visualization; supervision. All authors have approved the final version of the manuscript and agree to be accountable for the accuracy and integrity of the work.

## Conflict of interest statement

The authors declare no conflicts of interest.

## Code availability

The code developed during this study is available upon reasonable request. Analyses were performed using Stata, version 18 (StataCorp, College Station, Texas, USA).

## Prior presentations

Presented in part at the European Society of Medical Oncology Breast Cancer Congress, May 15–17, 2024, Berlin, Germany.

## Patient consent statement

As this study is a meta‐analysis of previously published research, no new patient consent was required.

## Supporting information




**Figure S1**: Funnel plot for disease‐free survival analyses
**Figure S2**: Galbraith plot for disease‐free survival analyses
**Figure S3**: Funnel plot for overall survival analyses
**Figure S4**: Galbraith plot for overall survival analyses
**Figure S5**: Funnel plot for breast cancer mortality analyses
**Figure S6**: Galbraith plot for breast cancer mortality analyses
**Figure S7**: Funnel plot for recurrence analyses
**Figure S8**: Galbraith plot for recurrence analyses
**Figure S9**: Trim‐and‐fill funnel plot of disease‐free survival
**Figure S10**: Forest plot for the association between metabolic syndrome and disease‐free survival including only studies assessed as of high quality using Newcastle‐Ottawa Scale
**Figure S11**: Forest plot for the association between metabolic syndrome and disease‐free survival including only studies assessed as of moderate or low quality using Newcastle‐Ottawa Scale
**Table S1**: Trim‐and‐fill analysis of disease‐free survival
**Table S2**: Newcastle‐Ottawa Scale (NOS) Scores for Included Studies

## Data Availability

The data underlying this article are available in the article and in its online Supporting Information.
