# Supplementary material for: Metabolic syndrome is associated with breast cancer mortality: A systematic review and meta‐analysis
Source: J Intern Med. 2025 Jan 8;297(3):262–75. doi: 10.1111/joim.20052 (PMC11846077; doi:10.1111/joim.20052)
Supplement: Supplementary file 1 — Figure S1: Funnel plot for disease‐free survival analyses Figure S2: Galbraith plot for disease‐free survival analyses Figure S3: Funnel plot for overall survival analyses Figure S4: Galbraith plot for overall survival analyses Figure S5: Funnel plot for breast cancer mortality analyses Figure S6: Galbraith plot for breast cancer mortality analyses Figure S7: Funnel plot for recurrence analyses Figure S8: Galbraith plot for recurrence analyses Figure S9: Trim‐and‐fill funnel plot of disease‐free survival Figure S10: Forest plot for the association between metabolic syndrome and disease‐free survival including only studies assessed as of high quality using Newcastle‐Ottawa Scale Figure S11: Forest plot for the association between metabolic syndrome and disease‐free survival including only studies assessed as of moderate or low quality using Newcastle‐Ottawa Scale Table S1: Trim‐and‐fill analysis of disease‐free survival Table S2: Newcastle‐Ottawa Scale (NOS) Scores for Included Studies [file JOIM-297-262-s001.docx]

**Figure S1 – Funnel plot for disease-free survival analyses**

###

**Figure S2 – Galbraith plot for disease-free survival analyses**

**Figure S3 – Funnel plot for overall survival analyses**

**Figure S4 – Galbraith plot for overall survival analyses**

####

**Figure S5 – Funnel plot for breast cancer mortality analyses**

####

**Figure S6 – Galbraith plot for breast cancer mortality analyses**

###

**Figure S7 – Funnel plot for recurrence analyses**

**Figure S8 – Galbraith plot for recurrence analyses**

**Disease-free survival (indicated due to significant Egger’s test)**

**Table S1 – Trim-and-fill analysis of disease-free survival**

**Figure S9 – Trim-and-fill funnel plot of disease-free survival**

**Table S2 – Newcastle-Ottawa Scale (NOS) Scores for Included Studies**

| **Study** | **Selection (max 4)** | **Comparability (max 2)** | **Outcome (max 3)** | **Total Score (max 9)** |
| --- | --- | --- | --- | --- |
| **Pasanisi P. (2006)** | **3** | **1** | **2** | **6** |
| **Bjørge T. (2010)** | **4** | **2** | **3** | **9** |
| **Oh S. (2011)** | **3** | **1** | **2** | **6** |
| **Berrino F. (2014)** | **3** | **1** | **2** | **6** |
| **Calip G. (2014)** | **4** | **2** | **3** | **9** |
| **Fan Y. (2015)** | **3** | **1** | **2** | **6** |
| **Cho W. (2018)** | **3** | **1** | **2** | **6** |
| **Gathirua-Mwangi W. (2018)** | **4** | **2** | **3** | **9** |
| **Grybach S. (2018)** | **3** | **1** | **2** | **6** |
| **Dibaba D. (2019)** | **4** | **2** | **3** | **9** |
| **Watanabe J. (2019)** | **3** | **1** | **2** | **6** |
| **Buono G. (2020)** | **4** | **2** | **3** | **9** |
| **Kennard K. (2021)** | **3** | **1** | **2** | **6** |
| **Taroeno-Hariadi K. (2022)** | **3** | **1** | **2** | **6** |
| **Yang P. (2022)** | **3** | **1** | **2** | **6** |
| **Zhou Z. (2023)** | **3** | **1** | **2** | **6** |
| **Chlebowski R. (2024)** | **4** | **2** | **3** | **9** |

**Figure S10 – Forest plot for the association between metabolic syndrome and disease-free survival including only studies assessed as of high quality using Newcastle-Ottawa Scale**

**Figure S11 - Forest plot for the association between metabolic syndrome and disease-free survival including only studies assessed as of moderate or low quality using Newcastle-Ottawa Scale**

**Meta-regression**

**1. Model Fit and Heterogeneity:**

**o Number of observations:** 10 studies included in the meta-regression.

**o Tau-squared (τ²):** 0.08111, indicating the estimated variance of the true effect sizes across studies.

**o I-squared (I²):** 48.97%, showing that approximately 49% of the variability in effect sizes is due to heterogeneity rather than sampling error.

**o H-squared (H²):** 1.96, which is another measure of heterogeneity.

**o Residual homogeneity test (Q_res):** The Q_res value is 9.76 with a p-value of 0.0824. This suggests that there is some residual heterogeneity that is not accounted for by the model, but it is not statistically significant at the 0.05 level.

**2. R-squared (R²):** 0.00%, meaning that the covariates (population, followup, age, and country) do not explain any of the heterogeneity in effect sizes.

**3. Wald chi-squared test:** The overall test of the regression model has a chi-squared value of 1.98 with a p-value of 0.7394. This indicates that, collectively, the covariates do not significantly predict the effect sizes.

**4. Coefficients and Interpretation:**

**o** Population: Coefficient is -5.24e-06 with a p-value of 0.918, suggesting no significant effect. The confidence interval (-0.0001046 to 0.0000941) includes zero.

**o** Followup: Coefficient is -0.0055682 with a p-value of 0.186, indicating no significant effect. The confidence interval (-0.0138127 to 0.0026764) includes zero.

**o** Age: Coefficient is 0.0188316 with a p-value of 0.476, suggesting no significant effect. The confidence interval (-0.0328965 to 0.0705596) includes zero.

**o** Country: Coefficient is 0.0642832 with a p-value of 0.715, indicating no significant effect. The confidence interval (-0.2802338 to 0.4088001) includes zero.

**o** Constant (_cons): Coefficient is -0.263789 with a p-value of 0.856, suggesting no significant effect. The confidence interval (-3.109176 to 2.581598) includes zero.

In summary, the meta-regression indicates that none of the covariates (population, followup, age, and country) significantly predict the effect sizes. The model explains none of the heterogeneity (R² = 0.00%), and there is some residual heterogeneity that is not statistically significant. The overall model is not statistically significant (Wald chi² = 1.98, p = 0.7394).

**Search string**

| Database | Search | Hits |
| --- | --- | --- |
| PubMed | ((("Breast Neoplasms"[Mesh]) OR (breast cancer[Title/Abstract])) AND ((((((((("Metabolic Syndrome"[Mesh]) OR ("Cardiometabolic Risk Factors"[Mesh])) OR (cardiometabolic risk[Title/Abstract])) OR (cardiometabolic risk factor*[Title/Abstract])) OR (MetS[Title/Abstract])) OR (metabolic syndrome[Title/Abstract])) OR (metabolic syndrome X[Title/Abstract])) OR (cardiometabolic syndrome*[Title/Abstract])) OR (metabolic disorder*[Title/Abstract]))) AND ((((((((((("Prognosis"[Mesh]) OR ("Survival"[Mesh] OR "Mortality"[Mesh] OR "Disease-Free Survival"[Mesh])) OR ("Recurrence"[Mesh])) OR (prognosis[Title/Abstract])) OR (survival[Title/Abstract])) OR (mortality[Title/Abstract])) OR (disease-free survival[Title/Abstract])) OR (recurrence[Title/Abstract])) OR (overall-survival[Title/Abstract])) OR (outcome[Title/Abstract])) OR (recurrence free survival[Title/Abstract])) | 296 |
| EmBase | ('breast cancer'/exp OR 'breast cancer*' OR 'breast tumor*' OR 'breast neoplasm*') AND ('metabolic syndrome x'/exp OR 'metabolic disorder'/mj OR 'cardiometabolic risk factor'/exp OR 'cardiometabolic risk factor' OR 'cardiometabolic disorder' OR 'metabolic syndrome x' OR 'metabolic syndrome' OR 'mets') AND ('prognosis'/exp OR 'survival'/exp OR 'mortality'/exp OR 'outcome' OR 'mortality' OR 'survival' OR 'prognosis' OR 'disease free survival' OR 'recurrence' OR 'recurrence free survival' OR 'overall survival') AND ('article'/it OR 'review'/it) | 723 |
